# Influence of Vitamin D Level on Oral Health Status in Adult Hypophosphatasia

**DOI:** 10.1111/jop.70039

**Published:** 2025-08-11

**Authors:** Florian Dudde, Dominik Fildebrandt, Paul Kock, Karin Petz, Ralf Smeets, Martin Gosau, Michael Amling, Thomas Beikler, Florian Barvencik

**Affiliations:** ^1^ Department of Osteology and Biomechanics University Medical Center Hamburg‐Eppendorf Hamburg Germany; ^2^ Department of Periodontics, Preventive and Restorative Dentistry University Medical Center Hamburg‐Eppendorf Hamburg Germany; ^3^ Department of Oral and Maxillofacial Surgery University Medical Center Hamburg‐Eppendorf Hamburg Germany; ^4^ Department of Oral and Maxillofacial Surgery, Division of Regenerative Orofacial Medicine University Medical Center Hamburg‐Eppendorf Hamburg Germany

**Keywords:** adult, hypophosphatasia, oral health, vitamin D

## Abstract

**Aim:**

Vitamin D deficiency is common in hypophosphatasia (HPP) patients. However, its impact on oral health is unclear. Therefore, the purpose of this study was to investigate the relationship between Vitamin D levels and oral health in adults with hypophosphatasia.

**Materials and Methods:**

In this retrospective study, oral health and bone metabolism in HPP patients were examined. The Decayed/Missing/Filled Teeth (DMFT) index, clinical attachment level (CAL), probing pocket depth (PPD), and periodontal screening index (PSI) were among the metrics used to evaluate oral health.

**Results:**

The average age of the 48 HPP patients in the study was 42.21 (±15.78) years. A mean Vitamin D level of 29 μg/L was used to divide the participants into two groups. Compared to patients with Vitamin D levels above 29 μg/L, those with levels below this threshold showed noticeably worse oral health, as evidenced by higher PSI, PPD, decayed tooth count, and periodontitis severity index.

**Conclusion:**

The results imply that adults with HPP who have low Vitamin D levels have worse oral health. To potentially improve oral health, it is crucial to diagnose and treat Vitamin D deficiency in HPP patients, as it is a known risk factor for periodontitis in the general population.

## 1 Introduction

A rare genetic metabolic disorder is called hypophosphatasia (HPP) [1]. The alkaline phosphatase liver/bone/kidney type (*ALPL*) gene, which codes for the tissue‐nonspecific alkaline phosphatase (TNSALP), is mutated in this disease [1]. An increased risk of fractures, arthralgias, and myalgias is the consequence of pathogenic variants in the *ALPL* gene, which impair TNSALP function and lead to mineralization disorders in bone metabolism [1, 2]. Additional symptoms include exhaustion, generalized pain, and, most importantly, a poor state of oral health (i.e., early loss of primary teeth, atraumatic tooth loss, and periodontitis) [3, 4, 5]. HPP can be classified into several subforms, including perinatal, infantile, adolescent, adult, odontohypophosphatasia, and pseudohypophosphatasia, depending on the severity of the condition and when it first manifests [1, 6].

The oral cavity is impacted in nearly all of these HPP subforms [7, 8]. Early research has already shown that a lower level of TNSALP and/or more *ALPL* mutations are associated with worse oral health, which in turn leads to higher pyridoxal‐5‐phosphate substrate levels [3, 5]. Along with atraumatic premature tooth loss, severe periodontitis was more common and DMFT indices were especially low [3, 5]. Additionally, Vitamin D deficiency is common in HPP patients [9]. According to Wiedemann et al. [9], up to 73.5% of HPP patients had Vitamin D deficiency. Because TNSALP's function is compromised in HPP, Vitamin D, a crucial hormone in the regulation of bone metabolism, is already disrupted [1, 2, 10].

However, other areas, like the oral cavity, are also impacted by this hormone's deficiency [10, 11]. It has been demonstrated that a lack of Vitamin D raises the risk of periodontitis in the general population [10, 11]. Since laboratory markers (i.e., *pyridoxal 5′‐phosphate* = PLP) on oral health in HPP have been investigated recently, the question emerges whether low Vitamin D levels also have an impact on adult HPP's oral health status [5]. However, little research has been done on the disease's effects on oral health due to the rarity of the disease.

Furthermore, there is currently no published research on the connection between HPP patients' oral health and Vitamin D levels. Therefore, this study's goal was to investigate the relationship between Vitamin D levels and oral health in adults with HPP.

## 2 Materials and Methods

### 2.1 Data Collection

HPP patients treated at the University Hospital Hamburg's Department of Osteology and Biomechanics and the Department of Periodontics, Preventive, and Restorative Dentistry from 2017 to 2023 were enrolled in this retrospective study. Data of the respective study population have already been published elsewhere [5]. The sample size for this study was not predefined through power analysis due to its retrospective nature. A genetically verified adult form of HPP and an HPP diagnosis based on the most recent standards established by the International Working Group on HPP were prerequisites for inclusion [5, 12]. While oral health data were gathered from digital records (Charly Program by Solutio, Holzgerlingen, Germany) in the Department of Periodontics, Preventive, and Restorative Dentistry, information on bone metabolism and clinical findings was taken from patient files in the Department of Osteology and Biomechanics. Systemic comorbidities (e.g., diabetes mellitus, autoimmune disorders, or other metabolic diseases) were not a basis for exclusion, reflecting the real‐world complexity of managing HPP patients. Other types of HPP and incomplete records were exclusion criteria. In total, 48 patients met the study's inclusion criteria.

### 2.2 Clinical Data

For every patient, baseline clinical characteristics were documented, including age, gender, body mass index (BMI), mutation type, and American College of Medical Genetics and Genomics (ACMG) classification. Alkaline phosphatase (AP), bone‐specific alkaline phosphatase (bAP), parathyroid hormone (PTH), PLP, Vitamin D, magnesium, copper, phosphate, calcium, and other laboratory parameters were also examined. In order to evaluate bone density, the results of dual X‐ray absorptiometry (DXA) scans were also examined [5, 13]. Bone mineral density, T‐scores, and *Z*‐scores are the formats in which DXA measurements are displayed. Two weeks after the initial HPP diagnosis and concurrent serum sample collection, the intraoral examination was performed.

### 2.3 Oral Health Status

Oral hygiene status was assessed through comprehensive dental examinations evaluating multiple parameters. These included the DMFT index (Decayed, Missing, and Filled Teeth), the number of present natural teeth excluding third molars, the Periodontitis Screening Index (PSI), probing pocket depth (PPD), clinical attachment level (CAL), and the severity grade of periodontitis. Additionally, patients were interviewed regarding the timing of their first permanent tooth eruption. All oral evaluations were conducted by experienced periodontists (OF, KP, TB), each with a minimum of 2 years of specialized training.

### 2.4 Vitamin D‐Level Distribution

The purpose of this study was to investigate the effects of vitamin D levels on oral health in adults with hypophosphatasia (HPP). Participants were divided into two groups based on their vitamin D levels, using a threshold of 29 μg/L. This cutoff was selected as it represented the mean vitamin D level within the study population. This approach allowed for a balanced comparison of oral health outcomes between participants with higher and lower vitamin D levels relative to the average value observed in this specific cohort. By choosing this data‐driven threshold, the study aimed to ensure internal consistency and enhance the statistical power of group comparisons.

### 2.5 Statistical Analysis

The statistical approach has been described elsewhere [5]. In brief, baseline patient characteristics were presented by descriptive analysis. Continuous variables with normal distribution are shown as mean ± standard deviation, while binary variables are displayed with absolute and relative frequencies. Comparisons of continuous variables were conducted using Student's *t*‐test, and binary variables were analyzed with the chi‐square test. Bar charts were employed as visual aids. A *p*‐value of less than 0.05 was considered statistically significant. Furthermore, the effect sizes were calculated by Cohen's *d*. All statistical analyses were performed using SPSS version 28.0 (IBM, Markham, Canada).

## 3 Results

### 3.1 Baseline

Data on the oral health and bone metabolism of 48 hypophosphatasia (HPP) patients were gathered for this study. Participants were 42 years old on average (±15.78). There were 39 women (81.3%) and 9 men (18.8%) in the group (Table 1). The ACMG system was used to classify the patients' HPP variants, with ACMG class 5 being the most prevalent (50%) (Table 1). Based on their average vitamin D levels (28.98 μg/L), participants were split into two groups: Group A had levels at or above 29 μg/L (*n* = 21), and Group B had levels below 29 μg/L (*n* = 27) (*p* < 0.001) (Table 1).

**TABLE 1:** Baseline characteristics (*n* = 48).

Variable	Total (*n* = 48)	Vitamin D ≥ 29 μg/L (*n* = 21)	Vitamin D < 29 μg/L (*n* = 27)	*p*	Cohen's *d*
Age	42.21 (±15.78)	43.86 (±16.27)	40.93 (±15.58)	0.529	0.183
Gender				0.149	
Male	9 (18.8)	2 (9.5)	7 (25.9)		
Female	39 (81.3)	19 (90.5)	20 (74.1)		
BMI (kg/m^2^)	24.35 (±5.86)	22.00 (±3.81)	26.17 (±6.56)	0.013	0.759
ACMG Class				0.502	
1	0 (0)	0 (0)	0 (0)		
2	0 (0)	0 (0)	0 (0)		
3	16 (33.3)	9 (42.9)	7 (25.9)		
4	8 (16.7)	3 (14.3)	5 (18.5)		
5	24 (50.0)	9 (42.9)	15 (55.6)		
PLP (μg/L)	87.10 (±68.31)	88.49 (±81.77)	63.33 (±40.18)	0.223	0.392
AP (U/L)	33.83 (±20.83)	29.85 (±12.75)	38.95 (±27.55)	0.135	0.389
bAP (U/L)	5.93 (±7.23)	5.05 (±3.67)	7.06 (±10.14)	0.346	0.258
Magnesium (mmol/L)	0.85 (±0.07)	0.89 (±0.07)	0.83 (±0.06)	0.003	0.857
Copper (μg/L)	1075.55 (±395.43)	1031.21 (±379.35)	1101.42 (±410.26)	0.604	0.178
Calcium (mmol/L)	2.37 (±0.12)	2.41 (±0.12)	2.32 (±0.11)	0.016	0.767
Phosphate (mmol/L)	1.14 (±0.26)	1.20 (±0.26)	1.07 (±0.24)	0.072	0.511
Vitamin D (μg/L)	28.98 (±15.39)	41.70 (±14.23)	19.09 (±6.32)	< 0.001	1.993
PTH (ng/L)	44.15 (±18.82)	41.26 (±17.18)	46.40 (±20.03)	0.354	0.274

*Note*: Data are presented as mean ± SD and/or percentage.

Abbreviations: ACMG, American College of Medical Genetics; AP, alkaline phosphatase; bAP, bone specific alkaline phosphatase; BMI, body‐mass‐index; PLP, pyridoxal phosphate; PTH, parathyroid hormone.

Parathyroid hormone levels were higher in patients with lower vitamin D levels (Group B) (Group A = 41.26 ng/L vs. Group B = 46.40 ng/L) (Table 1). Group B participants also had significantly lower levels of phosphate (Group A = 1.20 mmol/L vs. Group B = 1.07 mmol/L; *p* = 0.072) and calcium (Group A = 2.41 mmol/L vs. Group B = 2.32 mmol/L; *p* = 0.016) (Table 1). Table 1 shows that Group B had lower magnesium levels than Group A (Group A = 0.89 mmol/L vs. Group B = 0.83 mmol/L; *p* = 0.003).

Additionally, Group A had higher PLP levels and lower levels of bone‐specific phosphatase and alkaline phosphatase (Table 1). The BMI values of patients in Group B were significantly higher than those in Group A; with vitamin D levels below 29 μg/L (Table 1).

### 3.2 DXA‐Scan

Although these differences were not statistically significant, patients with vitamin D levels below 29 μg/L had lower bone mineral density values for the spine, left femur, and right femur than those with levels of 29 μg/L or higher (Table 2). The T‐score and the right femur's bone mineral density, however, were noticeably lower in Group B patients (Table 2). Furthermore, patients from Group B had lower Z‐scores and T‐scores for the left femur and spine (Table 2).

**TABLE 2:** DXA scan (SD).

Variable	Total (*n* = 48)	Vitamin D ≥ 29 μg/L (*n* = 21)	Vitamin D < 29 μg/L (*n* = 27)	*p*	Cohen's *d*
BMD SC (g/cm^2^)	1.14 (±0.22)	1.18 (±0.24)	1.08 (±0.17)	0.385	0.476
T‐score SC	−0.16 (±1.59)	0.20 (±1.71)	−0.65 (±1.31)	0.098	0.533
Z‐score SC	−0.03 (±1.33)	0.20 (±1.47)	−0.34 (±1.08)	0.180	0.405
BMD left femur (g/cm^2^)	0.95 (±0.15)	0.99 (±0.16)	0.90 (±0.13)	0.053	0.625
T‐score left femur	−0.49 (±1.10)	−0.20 (±1.16)	−0.86 (±1.00)	0.061	0.585
Z‐score left femur	−0.21 (±0.98)	−0.05 (±1.16)	−0.42 (±0.76)	0.215	0.357
BMD right femur (g/cm^2^)	0.94 (±0.14)	0.98 (±0.15)	0.89 (±0.11)	0.031	0.699
T‐score right femur	−0.57 (±1.00)	−0.28 (±1.01)	−0.96 (±0.88)	0.033	0.714
Z‐score right femur	−0.28 (±0.95)	−0.11 (±1.07)	−0.49 (±0.75)	0.190	0.416

*Note*: Data are presented as mean ± SD.

Abbreviations: BMD, bone mineral density; SC, spinal column.

### 3.3 Oral Health Status—DMFT

**FIGURE 1:**
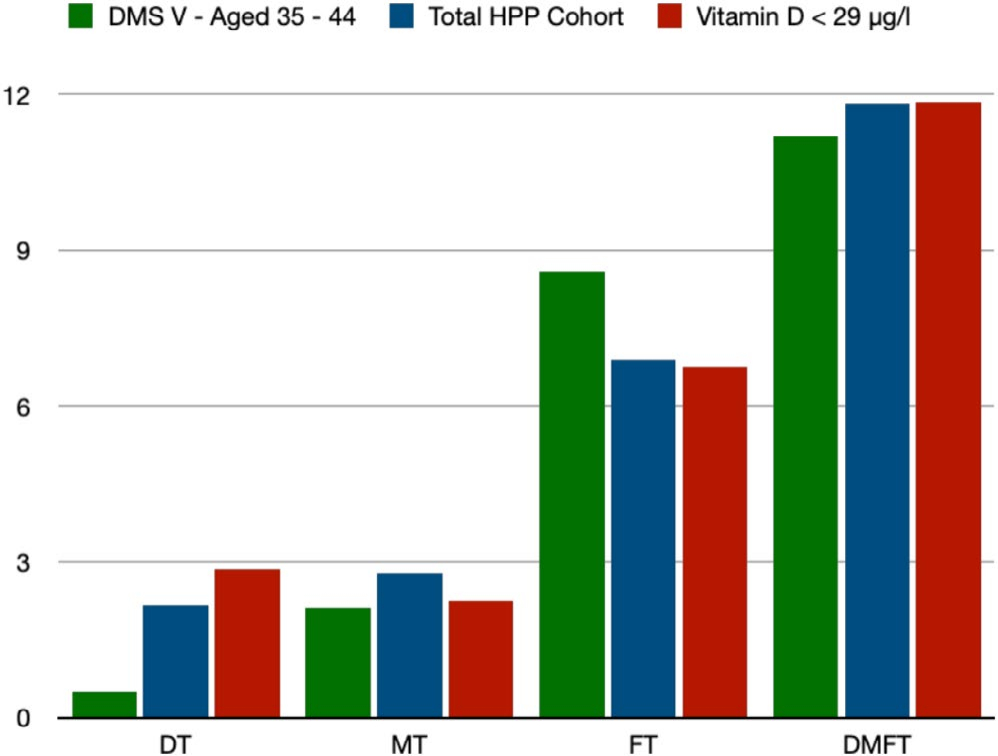
Bar plot—comparison of DMFT data with DMS‐V findings [14]. Data are presented as absolute values. DMFT, decayed, missing, filled teeth; DT, decayed teeth; FT, filled teeth; MT, missing teeth.

According to Table 3, patients with higher vitamin D levels also had more natural permanent teeth (Group A = 25.74 vs. Group B = 24.52). Additionally, patients with lower vitamin D levels had significantly more decayed teeth (Group A = 1.24 vs. Group B = 2.85; *p* = 0.021) (Table 3). Compared to Group B, patients in Group A had more missing and filled teeth (Table 3). Patients with lower vitamin D levels tended to have higher DMFT indices, even if there were no discernible differences between the two groups (Table 3, Figure 1).

**TABLE 3:** Oral health status—DMFT in HPP patients (SD).

Variable	Total (*n* = 48)	Vitamin D ≥ 29 μg/L (*n* = 21)	Vitamin D < 29 μg/L (*n* = 27)	*p*	Cohen's *d*
Number of natural permanent teeth	25.21 (±3.94)	25.74 (±3.23)	24.52 (±4.69)	0.293	0.299
Decayed	2.15 (±2.44)	1.24 (±1.76)	2.85 (±2.69)	0.021	0.684
Missing	2.79 (±3.94)	3.48 (±4.69)	2.26 (±3.23)	0.293	0.299
Filled	6.90 (±4.76)	7.10 (±5.44)	6.74 (±4.26)	0.801	0.007
DMFT‐Index	11.83 (±6.53)	11.81 (±7.25)	11.85 (±6.06)	0.983	0.005

*Note*: Data are presented as mean ± SD.

### 3.4 Oral Health Status—Periodontal Status

**FIGURE 2:**
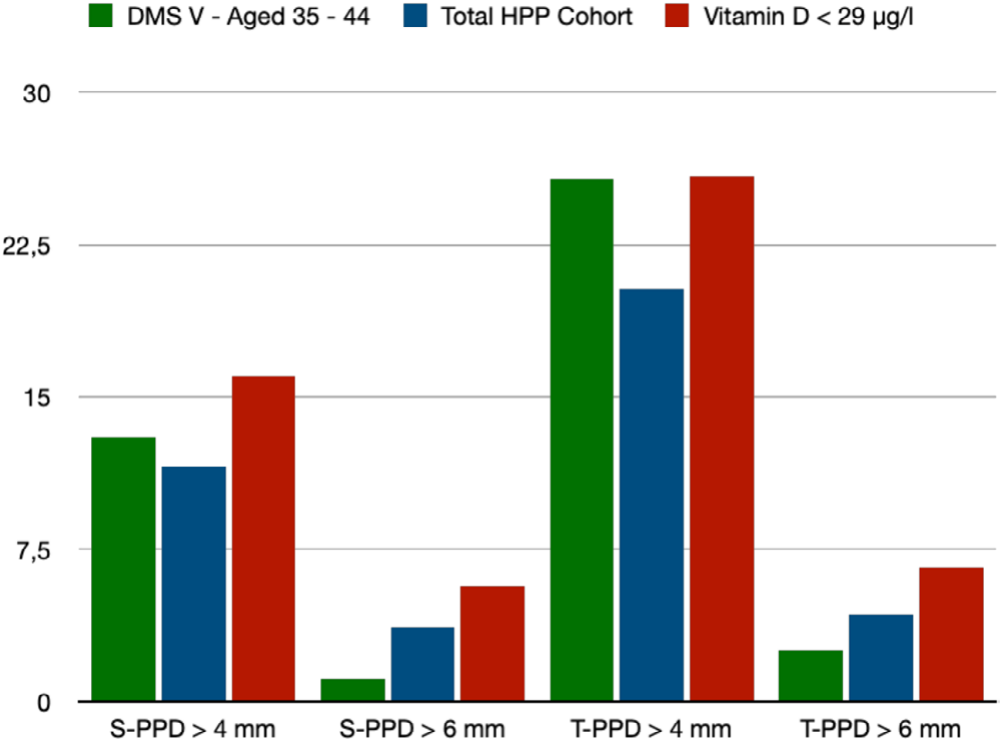
Bar plot—comparison of PPD data with DMS‐V findings [14]. Data are presented as a percentage. PPD, probing pocket depth; S, sides per tooth; T, teeth.

**FIGURE 3:**
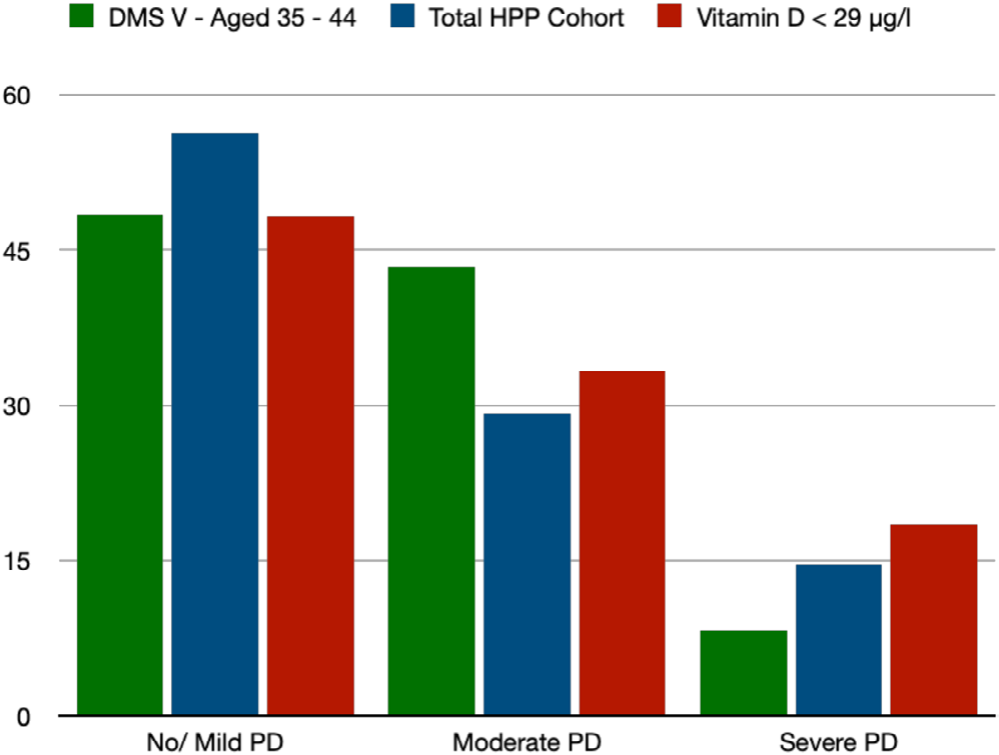
Bar plot—comparison of periodontitis severity grade with DMS‐V findings [14, 15]. Data are presented as percentages. PD, periodontitis.

PSI values were significantly higher in patients with a vitamin D level < 29 μg/L than in those with a level ≥ 29 μg/L (Table 4). With CAL > 3 mm and CAL > 5 mm, patients in Group B also displayed more sites and teeth per mouth (Table 4). In particular, there were highly significant differences between the sites and teeth per mouth with a PPD > 4 mm and PPD > 6 mm (Table 4, Figure 2). PPD > 4 mm and PPD > 6 mm were substantially more common in Group B patients than in Group A patients (Table 4). Patients with lower vitamin D levels had significantly higher rates of severe periodontitis (Group A = 9.5% vs. Group B = 18.5%; *p* = 0.042) according to the periodontitis severity grade (Table 4, Figure 3) [15]. Meanwhile, no/mild periodontitis was more common in patients with a vitamin D level ≥ 29 μg/L (Group A = 66.7% vs. Group B = 48.2%; *p* = 0.042) (Table 4).

**TABLE 4:** Detailed periodontal data (SD and/or percentage) in HPP patients.

Variable	Total (*n* = 48)	Vitamin D ≥ 29 μg/L (*n* = 21)	Vitamin D < 29 μg/L (*n* = 27)	*p*	Cohen's *d*
PSI–S1	2.19 (±1.02)	1.86 (±1.01)	2.44 (±0.97)	0.048	0.577
PSI–S2	1.94 (±0.93)	1.62 (±0.81)	2.19 (±0.96)	0.035	0.648
PSI–S3	2.13 (±1.04)	1.67 (±0.97)	2.48 (±0.98)	0.006	0.826
PSI–S4	2.04 (±0.97)	1.90 (±0.83)	2.15 (±1.06)	0.393	0.262
PSI–S5	2.10 (±1.03)	1.81 (±0.81)	2.33 (±0.73)	0.024	0.687
PSI–S6	1.92 (±1.03)	1.67 (±0.97)	2.11 (±1.05)	0.139	0.450
Sites per mouth CAL > 3 mm (%)	11.80 (±12.62)	11.11 (±12.15)	12.69 (±13.46)	0.673	0.123
Sites per mouth CAL > 5 mm (%)	2.31 (±6.10)	1.92 (±4.85)	2.80 (±7.51)	0.624	0.151
Teeth per mouth CAL > 3 mm (%)	13.10 (±12.09)	12.64 (±12.15)	13.68 (±12.29)	0.771	0.084
Teeth per mouth CAL > 5 mm (%)	2.38 (±5.41)	2.31 (±4.68)	2.47 (±6.35)	0.923	0.029
Sites per mouth PPD > 4 mm (%)	11.55 (±19.11)	5.84 (±10.36)	15.99 (±23.04)	0.047	0.596
Sites per mouth PPD > 6 mm (%)	3.69 (±9.70)	1.18 (±5.12)	5.65 (±11.87)	0.021	0.511
Teeth per mouth PPD > 4 mm (%)	20.30 (±24.09)	13.19 (±15.03)	25.83 (±28.34)	0.049	0.560
Teeth per mouth PPD > 6 mm (%)	4.29 (±11.36)	1.32 (±4.60)	6.61 (±14.29)	0.011	0.563
Age at first tooth exfoliation	5.06 (±0.63)	5.07 (±0.68)	5.05 (±0.59)	0.888	0.033
Periodontitis severity grade^a^				0.042	
No/mild PD	27 (56.3)	14 (66.7)	13 (48.2)		
Moderate PD	14 (29.2)	5 (23.8)	9 (33.3)		
Severe PD	7 (14.6)	2 (9.5)	5 (18.5)		

*Note*: Data are presented as mean ± SD and/or percentage.

Abbreviations: CAL, clinical attachment level; PD, periodontitis; PPD, probing pocket depth; PSI, periodontitis screening index.

^a^ Eke et al. [15].

## 4 Discussion

HPP is a rare inherited systemic metabolic disease with a variety of well‐defined systemic symptoms [2, 16]. In contrast to that, the affection of the oral cavity by HPP has only been superficially studied in the past. Therefore, there exists only scarce knowledge on the oral health status in HPP and the causal pathophysiology that leads to the observed dental and periodontal sequelae [3, 4, 5]. Since a recent publication with the same study population found that high PLP levels correlate with a negative oral health status in HPP patients, this analysis aimed to investigate the relationship between vitamin D levels and oral health in adult HPP [5].

Vitamin D deficiency is one of the most common vitamin deficiencies [17]. Vitamin deficiencies can manifest themselves in various symptoms, i.e., myopathies, rickets, and osteomalacia as an expression of dysregulated bone metabolism [18, 19]. Studies have shown that the deficiency of active vitamin D (Calcitriol) in animal models is associated with a loss of alveolar bone, increased gingival inflammation, and increased levels of proinflammatory cytokines (tumor necrosis factor‐α, matrix metalloproteinases 3/6, interleukin‐1β) [10, 20, 21]. These proinflammatory cytokines also play a crucial role in the development and modulation of periodontitis [22, 23, 24]. Grenier et al. [25] demonstrated that Calcitriol directly inhibits the growth of *Porphyromonas gingivalis* , one of the most common periodontitis germs, and also reduces the expression of other virulence factors (proteinases, adhesins). Since vitamin D fulfills other immunomodulatory functions (suppression of T‐lymphocytes, secretion of immunoglobulins, regulation of adaptive immune response), a deficiency of this vitamin consequently leads to an impairment of the systemic and specifically the local, i.e., oral immune response [10]. The impairment of the immune system was clinically supported by the fact that patients with a vitamin D level < 29 μg/L had significantly higher PSI values than patients with a vitamin D level ≥ 29 μg/L. Furthermore, patients with lower vitamin D levels exhibited significantly higher PPD than patients with higher vitamin D levels as an expression of an increased periodontal inflammation. Millen et al. [26] found similar results in a study of the effects of vitamin D levels on periodontal status in postmenopausal women. In the present study, patients with a lower vitamin D level showed a CAL > 3 and > 5 mm more often than patients with a higher vitamin D level; however, without statistical significance. Also, Millen et al. [26] could not demonstrate a significant correlation between the vitamin D level and the CAL in the context of an increased periodontal inflammation (higher PPD, higher Bleeding Indices). In direct comparison with the results of the German Oral Health Study‐V (DMS‐V) (age‐correlated healthy normal population aged 35–44), differences were found both for the entire study cohort and primarily for HPP patients with a vitamin D level < 29 μg/L (Figures 1, 2, 3) [14]. The DMFT index was increased in HPP patients compared to the DMS‐V cohort. HPP patients also revealed higher numbers of decayed and missing teeth compared to the DMS‐V study [14]. There were clear differences between HPP patients and the results of the DMS‐V study, particularly regarding the sites per tooth and teeth per mouth with a PPD > 6 mm (Figure 2) [14]. In addition, these differences were also evident regarding the severity of periodontitis [14, 17]. Patients with a vitamin D level < 29 μg/L had significantly worse periodontitis severity scores contrasted with patients showing a vitamin D level ≥ 29 μg/L [14, 17]. In comparison with the results of the DMS‐V study, all HPP patients and especially HPP patients with a vitamin D level < 29 μg/L were diagnosed with severe periodontitis at a higher frequency than would be expected in the age‐matched population [14].

In addition to the dental findings, patients with a vitamin D level < 29 μg/L showed significantly higher BMI values. The correlation of a high BMI value and lower vitamin D levels/vitamin D deficiency was also proven in studies by Lagunova et al. [27] and Kumaratne et al. [28]. Possible explanations for this correlation may be reduced sunlight exposure with simultaneously decreased participation in outdoor activities in patients with higher BMI values (i.e., obesity) as well as decreased expression of specific genes for vitamin D synthesis (i.e., 25‐hydroxylase in adipose and liver tissue) in obese patients consequently leading to lower serum vitamin D levels [29, 30].

Furthermore, patients with a vitamin D level < 29 μg/L revealed lower bone mineral density values, T‐scores, and Z‐scores for the spinal column as well as both femora; however, without statistical significance (Table 2). One possible biological plausibility for this observation arises from the deficiency of vitamin D, which is essential for the regulation of calcium levels (i.e., intestinal calcium absorption, renal calcium reabsorption) and the modulation of bone metabolism [31, 32]. Calcitriol indirectly promotes bone mineralization by increasing the calcium level (i.e., higher Calcium resorption, higher intestinal Calcium uptake) while simultaneously stimulating osteoblasts (i.e., synthesis of calcium‐binding proteins, synthesis of matrix metalloproteases for bone turnover) [31, 32]. Consequently, a vitamin D deficiency is associated with a lower calcium supply and reduced bone mineralization/reduced bone mineral density (Tables 1 and 2) [19]. Not surprisingly, a significantly lower serum calcium level was found in patients with a vitamin D level < 29 μg/L, as well as a reduced serum phosphate level with compensatory increased parathyroid hormone levels. Since bone metabolism is already disturbed in HPP due to the impaired function of TNSALP, an additional vitamin D deficiency may aggravate the detrimental effects on the bone as well as on dental and periodontal structures. In this regard, a previous study has shown that HPP teeth have severe hypomineralizations in the dentin and significantly reduced calcium content of the cementum [33]. Furthermore, Wölfel et al. [33] showed that HPP patients revealed a deficit of the acellular cementum with increased proportions of repair cementum thickness. Consequently, the periodontium in HPP is weakened by the reduced TNSALP enzyme activity with the above‐mentioned effects on dentin, cementum, and periodontal ligament. This may explain the worsening of the periodontal status by vitamin D deficiencies/reduced vitamin D levels (i.e., less immunomodulation, higher proinflammatory cytokines) [5, 10, 21, 25, 33].

While the primary focus of this study was on statistically significant findings, certain results, such as trends observed in BMD indicators, merit further consideration despite not achieving statistical significance. These trends may still provide valuable insights into potential relationships between vitamin D levels and systemic or oral health outcomes in HPP patients. Several plausible factors could explain these non‐significant trends. First, the relatively small sample size may have limited the statistical power to detect significant differences, particularly for variables with high inter‐individual variability. Additionally, confounding factors such as differences in age, disease severity, dietary habits, or baseline vitamin D levels may have influenced the observed trends but were not fully controlled in this retrospective analysis. From a biological perspective, the observed patterns may reflect the multifaceted role of vitamin D in bone and periodontal health [5, 10, 21, 25, 33]. Vitamin D's involvement in bone remodeling, mineralization, and immune regulation may contribute to subtle changes in BMD and periodontal outcomes that are not immediately detectable with statistical significance in a small cohort.

This study has several limitations. One of the limitations of this study is the inclusion of patients with systemic diseases that are known to influence periodontal health, such as diabetes mellitus, autoimmune disorders, and metabolic syndromes. While this approach reflects the real‐world clinical population of HPP patients, it introduces potential confounding factors that could influence the observed associations between vitamin D levels and oral health parameters. Although we documented systemic conditions and medications comprehensively, the absence of exclusion criteria for these factors limits our ability to isolate the direct effects of vitamin D levels and HPP on periodontal health. Future studies with stricter exclusion criteria or stratified analyses to account for systemic comorbidities are needed to validate and refine these findings. Another limitation of this study is the absence of effect size calculations to complement the statistical analyses. While *t*‐tests and chi‐square tests were used to evaluate group differences, reporting effect sizes (e.g., Cohen's *d* or Cramer's V) would have provided additional insights into the practical significance of the findings beyond *p*‐values. This omission may limit the interpretability of the magnitude of the observed differences.

A further limitation of this study is the absence of regression analysis to control for potential confounding factors and to evaluate the independent effects of vitamin D levels on oral health outcomes. While regression analysis could provide valuable insights, the relatively small sample size (*n* = 48) limited the feasibility of such an approach. Performing regression analysis with this cohort would have increased the risk of overfitting and reduced the reliability of the results.

Future studies with larger populations should incorporate regression models to better account for confounding variables and to provide a more robust assessment of the observed relationships. In addition to that, these studies should incorporate effect size calculations to enhance the understanding of the clinical relevance of group differences and to provide a more comprehensive statistical analysis.

Furthermore, it is a retrospective analysis with a relatively small sample size and is conducted at a single center without a follow‐up protocol. Consequently, the long‐term effects of hypophosphatasia and vitamin D deficiency treatment on oral health were not assessed.

However, Wiedemann et al. [9] were able to demonstrate that the general therapy recommendations for vitamin D repletion can also be applied safely in HPP without aggravating the underlying disease and/or worsening bone metabolism. Therefore, we conclude that vitamin D repletion may maintain and/or improve the oral health status in HPP patients. While this study highlights the potential importance of vitamin D levels in managing oral health outcomes in HPP patients, specific recommendations regarding supplementation dosage and monitoring protocols were not within the scope of this retrospective analysis. Data on patient‐specific supplementation regimens, including dosage and frequency, were not consistently available, limiting our ability to provide precise guidance on optimal strategies. Additionally, variability in individual factors such as age, weight, baseline vitamin D levels, and comorbid conditions further complicates the establishment of standardized recommendations. To address this gap, future research should focus on prospective interventional studies that explore the impact of different vitamin D supplementation regimens on oral health outcomes in HPP patients. Such studies could evaluate the efficacy of various dosages, determine the appropriate monitoring intervals, and identify specific thresholds for achieving optimal clinical benefits. Incorporating these elements into clinical management guidelines would enhance the practical applicability of research findings and improve patient care.

## 5 Conclusion

In conclusion, the present study revealed that low vitamin D levels are associated with poorer oral health status in adults with hypophosphatasia (HPP). Significant differences were observed regarding probing pocket depth, periodontitis severity, and the number of decayed teeth. As vitamin D deficiency is a known risk factor for periodontitis, adequate management of vitamin D levels may be essential for maintaining oral health not only in the general population but also in individuals with HPP. To build upon these findings, future prospective and interventional studies are warranted to evaluate the impact of targeted vitamin D supplementation on periodontal outcomes in HPP patients. Such research could inform clinical guidelines and lead to individualized prevention strategies aimed at improving long‐term oral and systemic health in this vulnerable population.

## Author Contributions

F.B., P.K., D.F., K.P., T.B., M.A. treated the patients and revised the article. F.D. researched the scientific literature, provided statistical findings/analysis, and wrote the article. R.S. and M.G. edited the manuscript. All authors gave final approval for publication.

## Ethics Statement

This study was conducted in accordance with the principles of the Declaration of Helsinki and approved by the University Medical Center Hamburg (UKE) in order to fulfill the requirements of a dental study thesis (FD) as well as the local ethics committee (MC 386‐18). All the procedures/diagnostics being performed were part of the routine care.

## Consent

All authors gave final approval for publication. All the procedures/diagnostics being performed were part of the routine care. Due to the retrospective analysis of the data, consent was waived. The authors affirm that human research participants provided informed consent for publication of the images/figures.

## Conflicts of Interest

F.B. received speakers fees and research grants from Alexion, UCB, and Diasorin, but not for this study. T.B. received speakers fees from Alexion. The other authors have no relevant financial or non‐financial interests to disclose.

## Data Availability

The data that support the findings of this study are available on request from the corresponding author. The data are not publicly available due to privacy or ethical restrictions.
